# Supplementary material for: Predicting visual function by interpreting a neuronal wiring diagram
Source: Nature. 2024 Oct 2;634(8032):113–23. doi: 10.1038/s41586-024-07953-5 (PMC11446822; doi:10.1038/s41586-024-07953-5)
Supplement: Supplementary file 6 — CRF and ERF predictions for individual TmY4 and TmY9 cells. Analogous to Supplementary Data 3, but for TmY target types. Shown are the top four monosynaptic pathways, the strongest pathway passing through each of the top ten intermediary types (ranking from Extended Data Fig. 7), and the trisynaptic pathway Tm1–TmY–Dm3–TmY (see the section entitled Prediction of spatial normalization). [file 41586_2024_7953_MOESM6_ESM.zip › DataS4/TmY9qΓèÑ/720575940627505027.pdf]

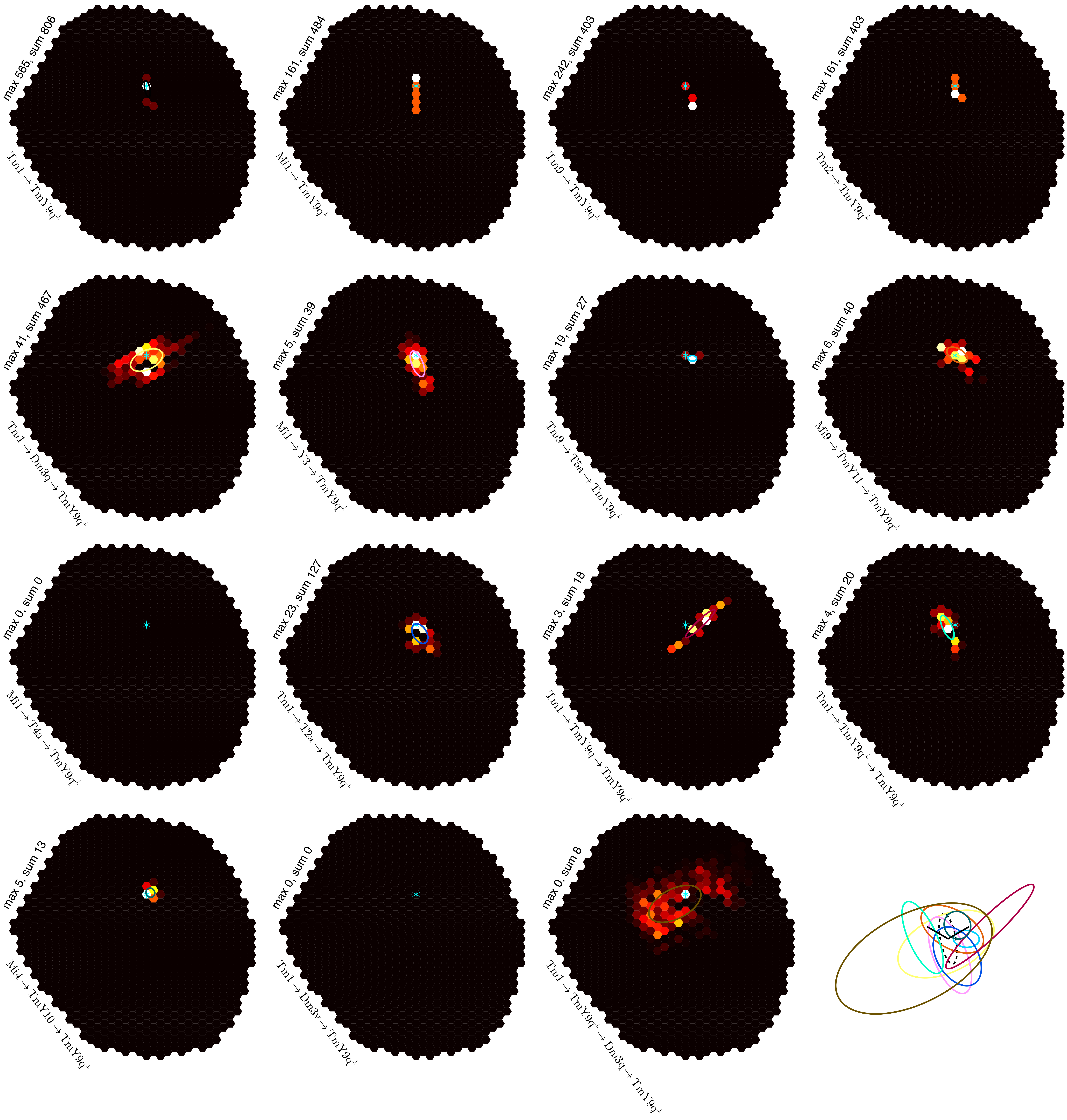
max 565, sum 806
Tm1 → $TmY9q^{\perp}$
max 161, sum 484
Mi1 → $TmY9q^{\perp}$
max 242, sum 403
Tm9 → $TmY9q^{\perp}$
max 161, sum 403
Tm2 → $TmY9q^{\perp}$
max 41, sum 467
Tm1 → Dm3q → $TmY9q^{\perp}$
max 5, sum 39
Mi1 → Y3 → $TmY9q^{\perp}$
max 19, sum 27
Tm9 → T5a → $TmY9q^{\perp}$
max 6, sum 40
Mi9 → TmY11 → $TmY9q^{\perp}$
max 0, sum 0
Mi1 → T4a → $TmY9q^{\perp}$
max 23, sum 127
Tm1 → T2a → $TmY9q^{\perp}$
max 3, sum 18
Tm1 → TmY9q → $TmY9q^{\perp}$
max 4, sum 20
Tm1 → $TmY9q^{\perp}$ → $TmY9q^{\perp}$
max 5, sum 13
Mi4 → TmY10 → $TmY9q^{\perp}$
max 0, sum 0
Tm1 → Dm3v → $TmY9q^{\perp}$
max 0, sum 8
Tm1 → $TmY9q^{\perp}$ → Dm3q → $TmY9q^{\perp}$
